# An industry perspective on Canadian patients' involvement in Medical Tourism: implications for public health

**DOI:** 10.1186/1471-2458-11-416

**Published:** 2011-05-31

**Authors:** Rory Johnston, Valorie A Crooks, Krystyna Adams, Jeremy Snyder, Paul Kingsbury

**Affiliations:** 1 Department of Geography, Simon Fraser University, 8888 University Drive, Burnaby, BC V5A 1S6, Canada; 2 Faculty of Health Sciences, Simon Fraser University, 8888 University Drive, Burnaby, BC V5A 1S6, Canada

## Abstract

**Background:**

The medical tourism industry, which assists patients with accessing non-emergency medical care abroad, has grown rapidly in recent years. A lack of reliable data about medical tourism makes it difficult to create policy, health system, and public health responses to address the associated risks and shortcomings, such as spread of infectious diseases, associated with this industry. This article addresses this knowledge gap by analyzing interviews conducted with Canadian medical tourism facilitators in order to understand Canadian patients' involvement in medical tourism and the implications of this involvement for public health.

**Methods:**

Semi-structured phone interviews were conducted with 12 medical facilitators from 10 companies in 2010. An exhaustive recruitment strategy was used to identify interviewees. Questions focused on business dimensions, information exchange, medical tourists' decision-making, and facilitators' roles in medical tourism. Thematic analysis was undertaken following data collection.

**Results:**

Facilitators helped their Canadian clients travel to 11 different countries. Estimates of the number of clients sent abroad annually varied due to demand factors. Facilitators commonly worked with medical tourists aged between 40 and 60 from a variety of socio-economic backgrounds who faced a number of potential barriers including affordability, fear of the unfamiliar, and lack of confidence. Medical tourists who chose not to use facilitators' services were thought to be interested in saving money or have cultural/familial connections to the destination country. Canadian doctors were commonly identified as barriers to securing clients.

**Conclusions:**

No effective Canadian public health response to medical tourism can treat medical tourists as a unified group with similar motivations for engaging in medical tourism and choosing similar mechanisms for doing so. This situation may be echoed in other countries with patients seeking care abroad. Therefore, a call for a comprehensive public health response to medical tourism and its effects should be coupled with a clear understanding that medical tourism is a highly diverse practice. This response must also acknowledge facilitators as important stakeholders in medical tourism.

## Background

Medical tourism occurs when patients intentionally leave their home countries for non-emergency medical care that is not part of a cross-border care arrangement [1-3]. In recent years, the global medical tourism industry has expanded greatly, with many thousands of patients from around the world traveling to countries near and far for medical care [3]. Hospitals around the world, and particularly those located in low and middle income countries, are offering an ever more comprehensive range of surgical and diagnostic procedures while they are increasingly active in seeking to attract international patients [4]. It is thought that patients seek medical care abroad because they: face long waiting lists in their home systems; want to access procedures that are illegal or unavailable at home; are seeking procedures not covered by a public health care system in their home country; or are uninsured or under-insured and are looking for an affordable care option [5-8].

Canadians are one of the patient groups known to travel abroad for medical care via the medical tourism industry [9]. While publicly financed provincial and territorial health care systems that offer universal coverage for essential surgeries operate in Canada, Canadians may opt to pursue care abroad for a number of reasons. It has been most commonly suggested that Canadian medical tourists are motivated by a desire to avoid surgical wait times [10]. However, Canadians may also be looking for less expensive options abroad for procedures that are not covered by their public insurance plan but are offered domestically, such as non-essential cosmetic procedures, or may be seeking to access novel procedures not at all available in Canada [1,8]. For Canadian patients engaging in medical tourism, this typically involves traveling abroad without a referral from a physician and paying out-of-pocket for treatments [8]. Canadians' involvement in medical tourism is, however, not limited to traveling abroad as consumers of care. Canadians have invested in destination hospitals abroad, and Canadian entrepreneurs have started facilitation companies that assist patients in securing care internationally [9,11,12].

In this article we explore the public health implications of Canadians' travel abroad as medical tourists from an industry perspective. A number of public health concerns for patients' home countries regarding medical tourism have been highlighted in the international literature. One is that there is little systematic data gathering and reporting in the medical tourism industry [1]. This makes it difficult to establish reliable numbers regarding patient involvement [5]. From a public health perspective, lack of data stymies efforts to create interventions that might maintain the health of medical tourists, or to create any monitoring of who is going abroad and where they are traveling to. A second public health concern is that in countries with publicly financed health care systems such as Canada, all citizens end up covering the costs of follow-up care and/or ongoing treatment and monitoring for those who choose to go abroad for medical care as medical tourists [13]. This issue has been raised when medical tourists return to their home countries with severe complications from surgeries abroad [13,14]. The costs of addressing these complications sometimes exceed the cost of having had the surgery done domestically. These circumstances demonstrate how public resources in patients' home countries can be burdened by medical tourism. A third public health concern is the potential for medical tourism to facilitate the global spread of infectious disease [15]. Medical tourism can involve people with weakened immune systems spending significant time in international hospitals. This may amplify the risk of disease transmission, as demonstrated in the case of New Delhi metallo-beta-lactamase (NDM-1) [15]. More specifically, in the latter half of 2010, eight Canadians contracted the NDM-1 'superbug', five of whom had contracted it while seeking medical care in India, all of whom required medical attention in Canada upon return [15]. Other concerns posed by medical tourism have also been regularly raised in discussions of the phenomenon, including the potential lack of legal recourse for injured patients, the uncertain quality of blood products and pharmaceuticals in some destination countries, and the heightened risk of post-operative embolisms posed by long haul travel [1,16].

In countries with established public health mandates such as Canada, it is widely thought that it is the responsibility of public health agencies and organizations to undertake measures to prevent infectious disease spread, maintain traveler health, monitor health risks, and ensure proper vaccination [17-19]-all of which are relevant to medical tourism. It is thus only logical to expect the issue of medical tourism to be on the 'radar' of such agencies and organizations. Recent years have confirmed an emerging awareness of medical tourism and the particular challenges it poses to public health initiatives within the public health communities of a number of medical tourists' home countries [20]. For example, the Public Health Agency of Canada and the Centers for Disease Control and Prevention in the United States have both released recommendations for patients considering medical tourism [18]. The development of these recommendation statements clearly demonstrates an emerging recognition within the North American public health community that medical tourism is a practice that warrants attention, if not concern.

The ongoing lack of evidence regarding medical tourism makes it difficult to create policy, health system, and public health responses to address any of the shortcomings of this practice [21]. In this article we contribute to addressing this knowledge gap through presenting an analysis of 12 interviews conducted with Canadian medical tourism facilitators. Facilitators are private operators used by some-but not all-medical tourists to assist with identifying doctors and hospitals abroad and making travel arrangements [22]. Here we draw on the findings of these interviews with the objective of characterizing several important, yet presently not well understood, dimensions of Canadians' travel abroad as medical tourists. Importantly, as discussed later on, the 10 companies represented by these 12 facilitators routinely have up to 1300 clients per year, which means that the findings shared here represent key trends among thousands of Canadians who have gone abroad for medical care via medical tourism in recent years. Using the findings, we ultimately move to consider the implications of Canadians' travel abroad as medical tourists for public health initiatives and professionals. To the best of our knowledge, this article offers the first insights into thinking about medical tourism as a *Canadian *public health issue, and in doing so contributes to the emerging international literature that is framing this global health care practice as a public health concern [1,20].

## Methods

This analysis contributes to an exploratory instructive case study that is seeking to understand Canadian patients' involvement in medical tourism.

### Recruitment

After obtaining ethical approval from Simon Fraser University's Office of Research Ethics, an exhaustive recruitment strategy was employed to identify medical tourism facilitation companies across Canada that operate using English. Companies were identified through published media accounts, reviewing Canadian membership in the Medical Tourism Association, and running searches in internet search engines. Internet searches involved exhaustively searching combinations of the keywords Canad*, medical, tour*, and surger* using Boolean operators. Twenty-two facilitation companies were identified using these strategies. A representative of each was contacted, requesting that one or more employees participate in an interview. Twelve individuals from 10 companies agreed to be interviewed.

### Data Collection

In mid-2010, semi-structured interviews were conducted by phone with medical tourism facilitators in all but one case where a face-to-face interview was requested. All but the face-to-face interview were conducted by the same interviewer, the fifth author, to enhance consistency. The face-to-face interview was conducted by the fourth author. Interviews lasted on average for 45 minutes.

Interviews were organized using a semi-structured guide. Semi-structured interviewing allows participants to add details beyond the specific questions being posed that they believe are relevant to the issues at hand [23]. Following a thorough review of the medical tourism literature to identify relevant issues, an interview guide was created that probed: business dimensions; information exchange with medical tourists; medical tourists' decision-making; and facilitators' roles in medical tourism. Selected questions from each section are included in Table 1.

**Table 1:** Selected Interview Questions

Topic	Example Questions
*Business Dimensions*	How long has the company been in operation for? Why did you decide to become involved in the medical tourism industry? How and where do you advertise your business?

*Information Exchange with Medical Tourists*	What types of information do you typically give potential medical tourists? Do you facilitate communication between doctors abroad and in Canada, or between potential medical tourists and doctors abroad?

*Medical Tourists' Decision-Making*	What are the different reasons that potential medical tourists have given you for wanting to seek care abroad? What role do you typically play with assisting potential medical tourists in their decision making regarding: (1) destination, (2) procedure, (3) physician/surgeon, (4) other factors? What role do you play in follow-up care?

*Facilitators' Roles in medical tourism*	What effects, positive or negative, does medical tourism have on Canada's health care system? Do you consider yourself to be an advocate for the patient, an advocate for the industry, and/or an advocate for the destination hospital/country?

### Analysis

Interviews were digitally recorded and transcribed verbatim. Transcripts were coded and managed using the N7™ software program. The first step in the analytic process was for each investigator to conduct independent transcript review. Following this, we collectively identified dominant issues emerging from the dataset that warranted in-depth analysis. Three meta-themes were identified, one of which pertained to trends and issues among Canadian medical tourists. It is this meta-theme that is examined in this article.

Following meta-theme identification, a thematic analysis was undertaken [24]. To accomplish this, a coding scheme was collaboratively developed using input from all investigators that was applied to the dataset. The first author then coded the data, which was verified by the third author. Following this, the first three authors reviewed coding extracts relevant to the meta-theme and developed an interpretive matrix-a grid that pairs discrete findings with specific sub-themes-to guide the thematic analysis [25]. Confirmation of the interpretive matrix was sought from all investigators. Consistent with a thematic approach to qualitative data [24], after the analytic findings pertaining to the meta-theme were determined the results were compared to the existing literature. Through this process a focus on the implications for public health was determined to be highly relevant to the findings. Our use of investigator triangulation throughout the analytic process in order to collaboratively identify analytic themes and confirm interpretation adds to the trustworthiness, and thus overall rigour, of the analysis [26].

## Results

The interviewed medical tourism facilitators were based in 3 Canadian provinces. The oldest company represented had been in operation for 13 years, and the youngest less than a year, with an average operation time of 4.5 years. All companies arranged for a number of different surgeries and treatments abroad, though typically a few were most favoured by each (e.g., orthopedic surgery, cosmetic surgery, fertility treatment).

In the remainder of this section we present the findings of the thematic analysis, with a focus on identifying trends and issues that characterize Canadian medical tourists from the perspectives of facilitators. Throughout we provide quotations that illustrate the findings, having used the standard process used by qualitative researchers of selecting quotes that best illustrate the issues at hand [23].

### Trends and Issues Related to Facilitators' Business Practices

**Figure 1:**
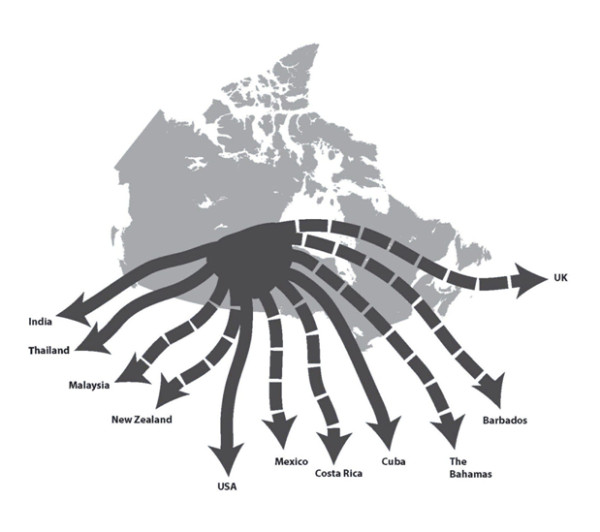
**Common Destinations Used By Canadian Medical Tourism Facilitators**. Figure 1 shows destination countries commonly used by the 12 interviewed medical tourism facilitators. Solid arrows indicate primary destinations. These are destinations that are used on a regular basis. Dashed arrows indicate secondary destinations. These are destinations that are used sometimes, though not infrequently. The United States of America (USA), Cuba, India, and Thailand are all primary destinations for 4 facilitators. Mexico and Costa Rica are the most common secondary destinations, each being used by 3 facilitators. New Zealand, Malaysia, Barbados, the United Kingdom (UK), and the Bahamas are each secondary destinations for 1 facilitator.

Facilitators assisted their clients with traveling to a range of countries, shown in Figure 1. Estimates of the number of Canadian medical tourists sent abroad annually to these countries by the 10 companies varied widely due to demand factors, collectively ranging from 1030-1335 per year. Five companies reported client loads of less than 50 per year, with three having less than 20 per year. Two had loads of between 50 and 200 a year, and three had more than 200. Many emphasized that their client loads had grown rapidly. For example, one remarked: "...we had about 50 patients in our first year...and double that in our second year...and in the third year we're coming close to doubling that again. So in the first three years, you know, we went from 50, to 100 to 180 patients" (Interviewee 3). An expanding client load was seen as a sure sign of future growth by facilitators.

Canadian medical tourists learned about facilitators and their services through two primary ways: word-of-mouth and internet searches. One facilitator succinctly captured the general consensus regarding the role of the internet when noting that: "We've tried a lot of different things and basically...the only thing that really works today is the internet, the web, and we've put a lot of investment into web pages" (Interviewee 3). The importance of word-of-mouth was demonstrated by another: "I get the most random referrals...you know, she [new client] was talking to someone...who had met somebody who had talked to me. Like it was three people away" (Interviewee 8). Less common methods of client recruitment included coverage of medical tourism and/or facilitators in news stories, trade fairs, and traditional media advertisements. Referrals from professional third parties were another less common source of clients, with insurance companies and doctors sometimes directly guiding patients to facilitators.

A wide spectrum of follow-up care practices were reported by the facilitators. The majority did not see their involvement extending to clients' aftercare. Of those that did involve themselves in follow-up care arrangements, a range of engagement was described. For example, one facilitator described taking a hands-off approach, in which they only provided follow-up care arrangements if specifically asked by the patient, while others would call clients upon their return home to prompt them to receive aftercare. The most involved end of the follow-up care spectrum was outlined as follows: "I won't set up the surgery and none of my partners will accept a patient until we have secured the post-care when they come back" (Interviewee 5).

### Trends and Issues Related to Medical Tourism Clients

The typical age of Canadian medical tourists was reported to be between 40 and 65, with fairly even representation from both employed and retired populations as well as both men and women. Clients' ages ranged from 18 months to 82 years. Some facilitators noted that there was a greater representation of younger people amongst those going abroad for cosmetic procedures. They worked with medical tourists from a variety of socio-economic backgrounds. However, most indicated that the majority of their clients came from middle socio-economic backgrounds with sufficient financial means to pay out-of-pocket for medical care.

**Figure 2:**
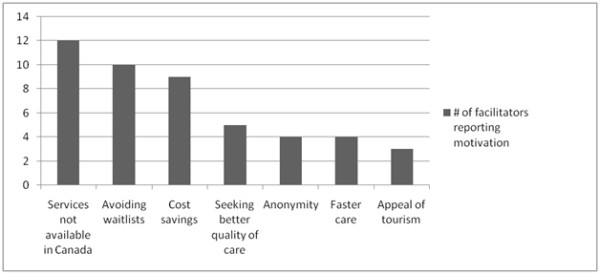
Motivations for Canadians' Engagement in Medical Tourism as Reported by Facilitators.

Facilitators explained that their medical tourist clients had typically received a diagnosis from a doctor and researched their desired procedure(s) prior to making contact with a facilitation company. Clients were prompted to go abroad for their desired procedure(s) for a number of reasons, summarized in Figure 2. One facilitator remarked: "We find that there are three classes of people who want to do medical tourism. There are those who want to save time. There are those who want to save money. And there are those who want treatments that are not available anywhere else in the world" (Interviewee 1). Many medical tourists were thought to be anxious not only about finding a way to improve their health, but also about finding solutions beyond those suggested by their doctors, which served to encourage their pursue of international care options.

Although medical tourists were characterized as being motivated to improve their health status, thus prompting them to consider accessing care abroad, they were often described by facilitators as scared by their health situation, unhappy with their current quality of life, and unsure about the idea of traveling abroad for medical care. This left facilitators needing to build up clients' confidence in going abroad for desired procedure(s), thus helping them to feel comfortable about their decision to leave Canada for medical care. A facilitator described this, saying: "It's a big you know leap of faith... A lot of people will ask a bunch of questions and then a year later they call us back and say 'alright well I'm ready to go now'... So there, there is definitely that sort of learning curve, where it's a confidence curve, people start out by saying, 'Where? India? You've got to be crazy!' And, um, then after awhile after, you know, they find out more about it and their confidence...grows" (Interviewee 2).

### Trends and Issues Related to Non-Utilization

Facilitators identified a number of potential barriers to going abroad for Canadians who are contemplating medical tourism. Financial barriers were commonly cited as reasons people refrain from pursuing medical tourism. Fear of the unfamiliar and a lack of confidence were other identified barriers. Canadian doctors were regularly cast as antagonists, with six facilitators identifying discouragement from family doctors and specialists as a major barrier to going abroad. One facilitator described this common view, saying: "...the biggest barrier to Canadians accessing treatment abroad, whether its abroad in the next town or abroad in the next province or across the globe...is their treating physician" (Interviewee 1). Some cited examples of space 'suddenly' being made in waiting lists by doctors of those seriously considering medical tourism: "[sometimes] once your treating physician finds out about it [plans to go abroad for medical care], a space miraculously opens up on the waiting list" (Interviewee 1). It was speculated that this was arranged for by clients' doctors to prevent them from exiting the public system. Less commonly identified reasons for not going abroad included: the client dying or becoming too ill to travel; the client being talked out of medical tourism by friends and family; space in the public system opening up naturally; and poor destination country reputations.

Participants spoke about why Canadians interested in medical tourism might not use a facilitator. Some believed this happened because patients wanted to save money by arranging for care on their own. Another related reason is that some medical tourists have existing cultural and/or social connections to the destination country, and would thus feel comfortable navigating its health care system on their own. For example, a participant explained that it was not uncommon for prospective clients of Indian heritage to consult with a facilitator for "information gathering" because they already "have relatives, family back home in India" who can help them with the logistics while abroad (Interviewee 9). A third reason that medical tourists might not use a facilitator is that Canadians were thought to be culturally unfamiliar with the idea of trusting business-people to oversee their medical care, unlike those exposed to a private system, and would therefore be disinclined to seek help in arranging for care abroad.

## Discussion

The findings shared above provide a first glimpse into the demographic makeup of Canada's medical tourist population, in addition to revealing key aspects of medical tourism facilitators' business practices and the mindsets of Canadians thinking about going abroad for medical care. The insights gleaned with regard to destinations utilized by Canadian medical tourists, standards of care with regard to post-operative guidance, and relationships with clients' physicians described by the medical tourism facilitators we interviewed raise a number of important implications for public health. In this section we expand upon these implications, considering their impacts both within and beyond Canada. Following this, future public health research directions related to medical tourism facilitation are suggested.

### Implications for Canadian Public Health

While our interviews with medical tourism facilitators provide a preliminary sense of the numbers of Canadians engaging in medical tourism and their destinations, systematic monitoring is needed in order to create a comprehensive picture. Such monitoring is necessary so as to develop health system responses and guard against infectious disease spread given that these are expressed public health concerns, as noted in the background section [5,13,15]. Importantly, the findings shared above demonstrate that while systematic monitoring of medical tourism by public health officials through facilitation companies may be useful, it would render only a partial picture as many Canadian medical tourists do not use their services. This is, however, not to suggest that facilitators should not be considered a rich source of data for surveillance efforts.

Worries about infectious disease being spread by medical tourists are common in public discussions of the medical tourism industry in a number of countries, including Canada [1,15]. This is a viable worry given that a number of the primary and secondary destination countries used by the facilitators we interviewed, shown in Figure 1, have active travel health notices from Public Health Agency of Canada (e.g., Dengue fever in Thailand and India, polio in India, malaria across much of the Global South) [27]. While the recommendations created by this same Agency for those considering accessing medical care in another country warn that standards of prevention against infectious disease may be different abroad [17], it is currently not clear whether or not medical tourists are being exposed to these concerns via facilitators. Given the important role that facilitators play in relation to Canadians' involvement in medical tourism, as demonstrated by our findings, it may be advisable to create informational interventions that target this stakeholder group. This could include creating facilitator recommendations along the lines of the Public Health Agency of Canada's patient recommendations, which could identify issues of disease transmission risk and possibly suggest ways of informing clients about how to minimize this risk.

Public health concerns related to medical tourism that have been given little existing attention have emerged from this study. One has to do with the role of health professionals as a conduit to needed health information for medical tourists. The medical tourism recommendations developed by the Public Health Agency of Canada currently suggest that patients discuss "your medical care plans with your health care provider before leaving and follow up when you return" [18]. This sound advice is complicated by the seemingly antagonistic relationship between facilitators and clients' doctors, as discussed during the interviews. Many facilitators indicated that they had difficulty communicating with clients' doctors, and that in some cases doctors worked actively to dissuade patients from engaging in medical tourism. This antagonism may spill over to the ongoing relationship between medical tourists and their doctors as well, though further research is required in order to determine whether or not this is the case. While doctors may have legitimate concerns about medical tourism, a troubled doctor-patient relationship may create a barrier to receiving advice regarding vaccines and other preventative or precautionary measures, obtaining prescriptions for medications required abroad, or arranging for follow-up care [1,28].

Further to the above, a second public health concern that has emerged from this study pertains to medical tourists' follow-up care upon return to Canada. The importance of medical tourists making sound follow-up care arrangements has become increasingly clear in recent months, with a medical tourist from the province of Ontario dying after complications from an experimental surgery that could not effectively be treated in Canada [29]. Clearly, facilitators have a role to play in ensuring that medical tourists make arrangements prior to traveling abroad for follow-up care upon return home. While they are usually not health professionals and so are not in a position to make referrals or appointments for services within Canada's public health care system, they are well positioned to prompt patients regarding the need to arrange follow-up care. The wide spectrum of follow-up care practices described, weighted towards non-involvement, suggest that this is another point that can be touched upon in public health outreach to Canadian medical tourism facilitators so as to work towards establishing a best standard of practice.

### Implications for Public Health beyond Canada

The public health concerns identified in this analysis, those of pre-trip preventative measures and preparation for international travel and surgery, patient flow and surveillance, infectious disease transmission, and appropriate and organized follow-up care, are not limited to the Canadian context. Each of these issues has the potential to impact medical tourists traveling from and/or to any number of countries. As such, it would behoove public health agencies and organizations in countries with identified outflows of patients who are traveling abroad as medical tourists to develop interventions that will ensure traveler and public health. In the previous sub-section we identified interventions that may be considered for adoption in these other countries, such as the development of a surveillance system that integrates information from facilitators and the creation of a facilitator recommendations statement that articulates health risks and safety measures, among other points of information.

Our primary consideration in this article has been on the public health implications for Canada as a country from which medical tourists depart. Though not our focus, consideration must be given to the public health implications of medical tourism on the countries that international patients are travelling to for medical care. One such implication pertains to the potential transformation of the health care system through a shifting of resources away from primary health care services, including disease prevention and health promotion efforts, and toward costly tertiary care services needed by hospitals wishing to attract international patients [30]. Any shift in funding and resources away from disease prevention and health promotion efforts may have a grave effect on public health mandates and health equity [4,31]. While discussion regarding infectious disease spread via medical tourism tends to focus on the spread of disease to international patients, it is not ill conceived to think that spread could occur in the opposite fashion, with citizens and health care providers being exposed to disease carried by international patients [20]. Public health agencies in destination countries need to take measures to minimize and also monitor any infectious disease spread through medical tourism [15].

### Future Research Directions

This analysis demonstrates that medical tourism facilitators are rich sources of information with valuable insights into the particular nature of local trends and issues surrounding medical tourism. As medical tourism facilitators are found in the home countries of medical tourists outside of Canada, it would be a valuable exercise for a similar analysis to be performed elsewhere. While facilitators cannot provide fully comprehensive accounts of medical tourism trends, discovering what they know in different regional contexts can begin to address the wide gap that exists in our current knowledge of medical tourism [1,5]. This could help inform, perhaps even spur, future public health surveillance and/or other interventions.

Medical tourism facilitators do not only operate in the home countries of medical tourists. There are facilitation companies operating in destination countries that serve international patients from a number of countries [22]. For example, a Canadian could employ the services of a facilitator working in Thailand to assist them with accessing care abroad either in Thailand or any other countries the facilitator refers clients to. Comparing and contrasting who uses these companies and for what services could further illuminate the nature of medical tourism. The public health concerns for Canada and other home countries for medical tourists raised in this study may also be confirmed, challenged or supplemented by speaking with medical tourism facilitators operating in destination countries themselves.

### Limitations

Our restriction to English-language data collection means that we may have excluded Canadian medical tourism facilitation companies operating in Quebec exclusively in French. While we are not aware of the existence of any such companies, they may exist nonetheless. These companies would not have been captured in our English-language search for potential interview candidates or may have felt uncomfortable replying to our request for an interview. Another limitation was our reliance on the internet to identify potential participants. Medical tourism facilitators that do not have a web presence would not have been contacted. Furthermore, the medical tourism facilitators we spoke with may have represented a particular niche in the Canadian medical tourism landscape. The companies that agreed to speak with us typically had more advanced websites than those that did not, which may be an indication of self selection amongst more marketing-conscious, networking-oriented facilitators. This potential for a self selection bias may have excluded less organized or engaged facilitation companies that might have provided different insights into the state of medical tourism facilitation in Canada. Finally, our reliance on phone interviewing serves as a limitation in the study design, whereby body language and emotions were not able to be captured during data collection.

## Conclusions

To the best of our knowledge, this study represents the first research engagement with medical Canadian tourism facilitators to discover what they know of the medical tourism phenomenon. These facilitators have proven to be a rich source of information about who Canada's medical tourists are, and who they are not. The findings of 12 interviews with Canadian medical tourism facilitators suggest that medical tourism is an industry of considerable size in Canada, engaged in by a heterogeneous group of clients, and perceived to be growing by those deeply engaged in the industry. Facilitators described a range of personal business practices related to the care of their clients and their exposure to risk. The examples of inconsistencies in these business practices provided by facilitators raise public health concerns around the involvement of family physicians in the planning of care abroad, follow-up care provided to medical tourists, and the prevention of infectious disease and surgical complications.

This study shows that no effective Canadian public health response to medical tourism can treat medical tourists as a unified group with similar motivations for engaging in medical tourism and choosing similar mechanisms for doing so. Those Canadian medical tourists who use facilitators are extremely varied in their characteristics and, as our findings have shown, many medical tourists do not engage with facilitators at all. Therefore, a call for a comprehensive public health response to medical tourism and its effects should be coupled with a clear understanding that medical tourism is a highly diverse practice. This response must also acknowledge facilitators as important stakeholders in Canadian medical tourism.

## List of Abbreviations Used

NDM-1: New Delhi metallo-beta-lactamase-1

## Competing interests

The authors declare that they have no competing interests.

## Authors' contributions

RJ coded the interview data, participated in the identification and organization of themes, and led drafting the paper. VAC conceived of and designed the study, participated in the identification of themes, and participated in drafting the paper. KA participated in the identification of themes and the drafting of the paper. JS participated in the identification of themes and the drafting of the paper. PK conducted the interviews, participated in the identification of themes, and reviewed the draft. All authors have reviewed and approved the submitted manuscript.

## Pre-publication history

The pre-publication history for this paper can be accessed here:

http://www.biomedcentral.com/1471-2458/11/416/prepub
